# Cough, sarcoidosis and idiopathic pulmonary fibrosis: raw nerves and bad vibrations

**DOI:** 10.1186/1745-9974-9-9

**Published:** 2013-03-06

**Authors:** Nicholas Kim Harrison

**Affiliations:** 1 College of Medicine, Swansea University, Swansea, Wales, UK

## Abstract

Cough is a common symptom in people who develop interstitial lung diseases (ILD). The pathological features of the ILDs are many and varied suggesting that the cause of cough may also vary with each disease. This article reviews what is currently known about cough in sarcoidosis and idiopathic pulmonary fibrosis; two of the commonest ILDs. It also outlines some of the theories which have been proposed to explain why cough develops in these conditions and describes what little is known about how to treat it.

## Sarcoidosis

Sarcoidosis is a multi-system disorder of unknown aetiology which is characterised histologically by the infiltration of affected tissue by non-caseating granulomatous inflammation. Whilst any part of the body can be involved, sarcoidosis typically affects hilar and mediastinal lymph nodes, the lung interstitium and bronchial epithelum. Less commonly, the upper respiratory tract, pleura and pulmonary vessels may be involved. A number of epidemiological studies have investigated the prevalence of cough in people with sarcoidosis and whilst it appears rare in Japan, it is common in other parts of the world where the prevalence is between 30 and 50% (Table 1) [1-3].

**Table 1:** Epidemiological studies of the prevalence of cough in sarcoidosis

**Authors (year)**	**n =**	**Country**	**Mean age**	**% Female**	**% Cough**
Pietinalho (1996) [1]	571	Finland	41	59%	33%
Pietinalho (1996) [1]	686	Japan	30	55%	3%
Kiter (2011) [2]	293	Turkey	44	67%	52%
Al-Khouzaie (2011) [3]	33	Saudi Arabia	44	54%	48%

(References 1–3).

**Figure 1:**
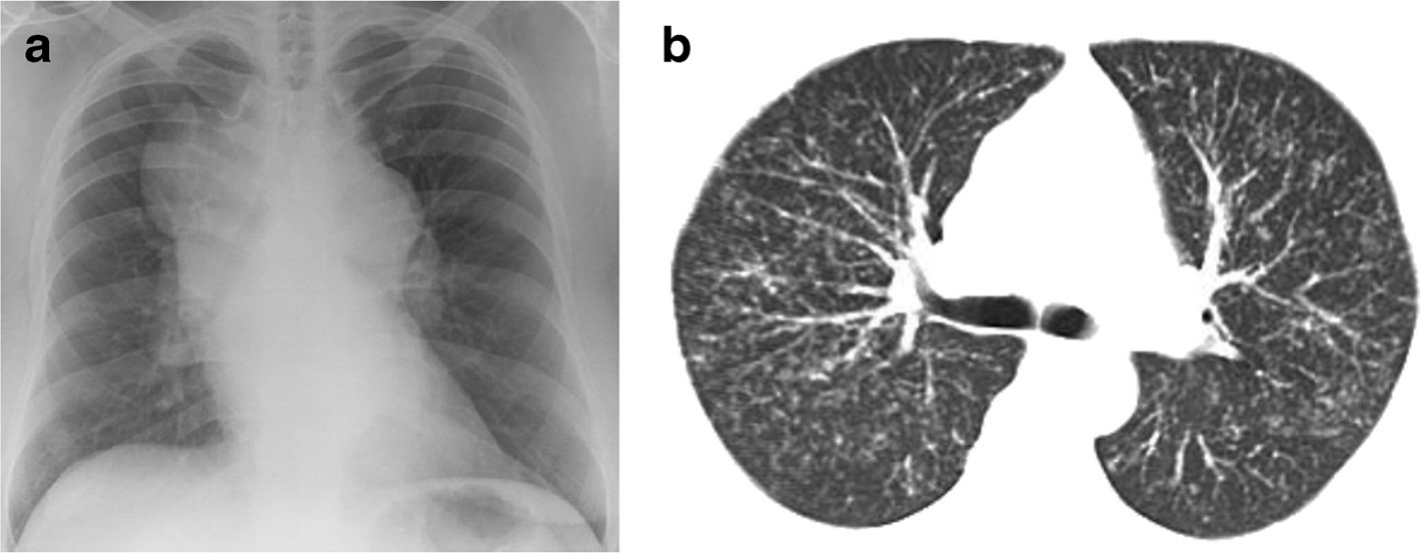
**a Chest radiograph showing massive hilar and mediastinal lymphadenopathy and b, thoracic CT scan showing diffuse nodular infiltration of the lung interstitium.** Neither of these patients complained of cough.

**Figure 2:**
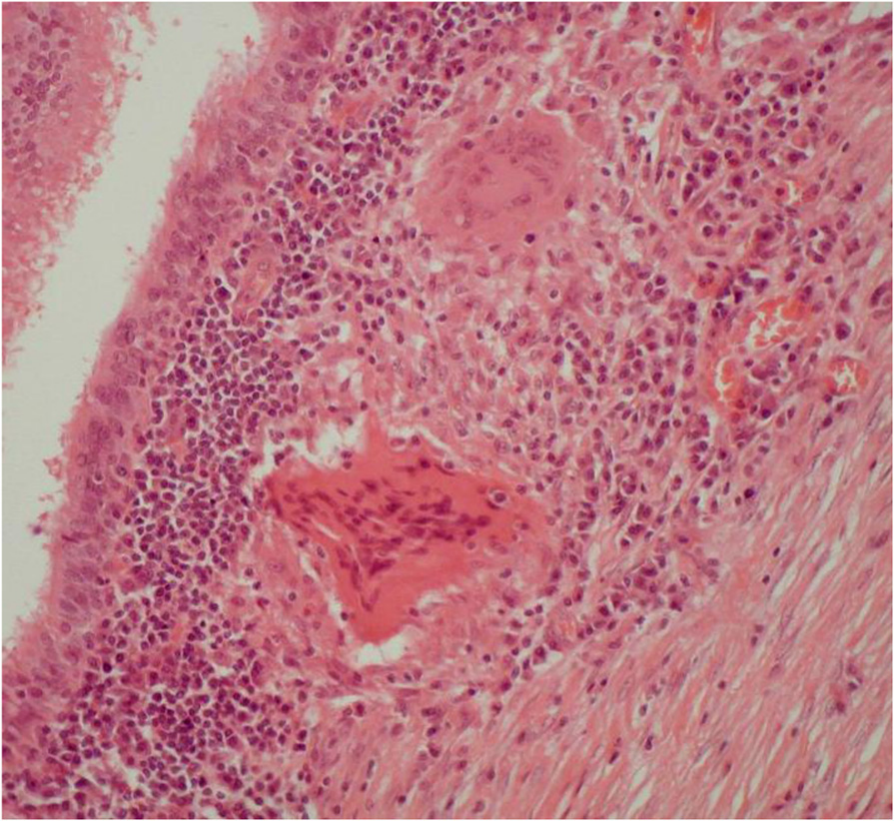
**Bronchial biopsy from a patient with sarcoidosis showing typical sub-mucosal granulomata (Haemotoxylin & Eosin x 20).** Photomicrograph courtesy of Drs Allan Dawson and Paul Griffiths.

Whilst little is known about the mechanisms by which sarcoidosis causes cough [4], clinical observation suggests that neither massive hilar and mediastinal lymphadenopathy nor interstitial infiltration are, in themselves, sufficient to cause significant cough (Figure 1). Perhaps more important is the recognition that sarcoidosis is often a bronchocentric disease associated with granulomatous inflammation of the airways [5]. This is not always apparent on radiological imaging and is best appreciated by fibreoptic bronchoscopy although it may sometimes only be detected by histological examination of endobronchial biopsies which otherwise have a normal macroscopic appearance (Figure 2).

One retrospective study from China supports the link between granulomatous inflammation of the airways and cough in sarcoidosis. This study compared respiratory symptoms in 87 patients who had undergone tracheal biopsy and found that significantly more (92%) of those who had granulomatous tracheitis complained of cough compared with those (49%) who had normal tracheal biopsies [6]. This study did not report on the bronchoscopic appearances of the bronchial mucosa nor on the histological appearances of bronchial biopsies but it seems plausible that cough in the latter group may have been caused by inflammation of the bronchi or smaller airways without tracheal involvement.

Sarcoidosis of the upper respiratory tract (SURT) occurs in approximately 5% of cases and may variably affect the larynx, epiglottis, arytenoids, the aryepiglottic folds and vocal chords as well as the nasal mucosa and sinuses [7]. There are no epidemiological studies of cough in relation to SURT but given the sensitivity of the glottis to mechanical and chemical stimulation in normal subjects it seems likely that inflammatory change in and around the larynx would increase cough reflex sensitivity.

The enhanced cough reflex observed in people with saroidosis is likely initiated by sensory fibres of the vagus nerve that innervate the airways and larynx which have become sensitised by granulomatous inflammation. The precise mechanism by which this occurs is uncertain. However, it is interesting that Ricci and co-workers demonstrated that a number of non-neuronal cell types can express neurotrophins and their receptors in normal human lung [8]. These include bronchial and alveolar epithelial cells, bronchial glands and mesenchymal cells of the interstitium as well as lymphocytes and macrophages. These observations led them to suggest that neurotrophins may play a role in the regulation of normal lung cell function and potentially, in the pathophysiology of lung disease [8]. In a subsequent study they demonstrated that alveolar macrophages and lymphocytes derived from bronchoalveolar lavage fluid from patients with sarcoidosis showed enhanced expression of neurotrophins and their receptors [9]. These findings were confirmed by Dagnell and coworkers who went on to show that sarcoid granulomas and alveolar macrophages stained for the neurotrophins Nerve Growth Factor (NGF), Brain-Derived Neurotrophic Factor (BDNF) and Neurotrophin-3 (NT3) as well as their receptors TrkA, TrkB and TrkC [10,11]. Thus, increased levels of neurotrophins occur at sites of granulomatous inflammation in the airways of patients with sarcoidosis - a location where they could potentially modulate sensory nerve proliferation and/or differentiation to enhance neuronal sensitivity and cause cough.

Occasional, unusual manifestations of sarcoidosis have been described which cause patients to present with the symptom of cough. These include granulomatous infiltration of the pulmonary veins causing pulmonary veno-occlusive disease with engorgement of airway epithelium and sarcoidosis affecting the vagus nerve at the level of the jugular foramen causing vocal cord palsy and cough [12,13].

There have been no randomised, placebo-controlled clinical trials which have investigated the effect of any pharmacological treatment specifically for cough in sarcoidosis. However, clinical observation suggests that when used in an appropriate clinical setting such as progressive interstitial lung disease, oral corticosteroid therapy has a beneficial effect on cough when it is a troublesome accompanying symptom. One double-blind, placebo-controlled study of inhaled corticosteroids in newly diagnosed patients with pulmonary sarcoidosis reported an improvement in global symptom score which included cough [14]. However, the placebo group were more symptomatic at the start of the study and the authors reported that both cough and fatigue persisted in a number of patients on active treatment as well as placebo. Thus whilst accepting the evidence is weak, a systemic review of steroid therapy in sarcoidosis has suggested that inhaled corticosteroids could be offered to selected patients with cough [15].

### Idiopathic pulmonary fibrosis

**Figure 3:**
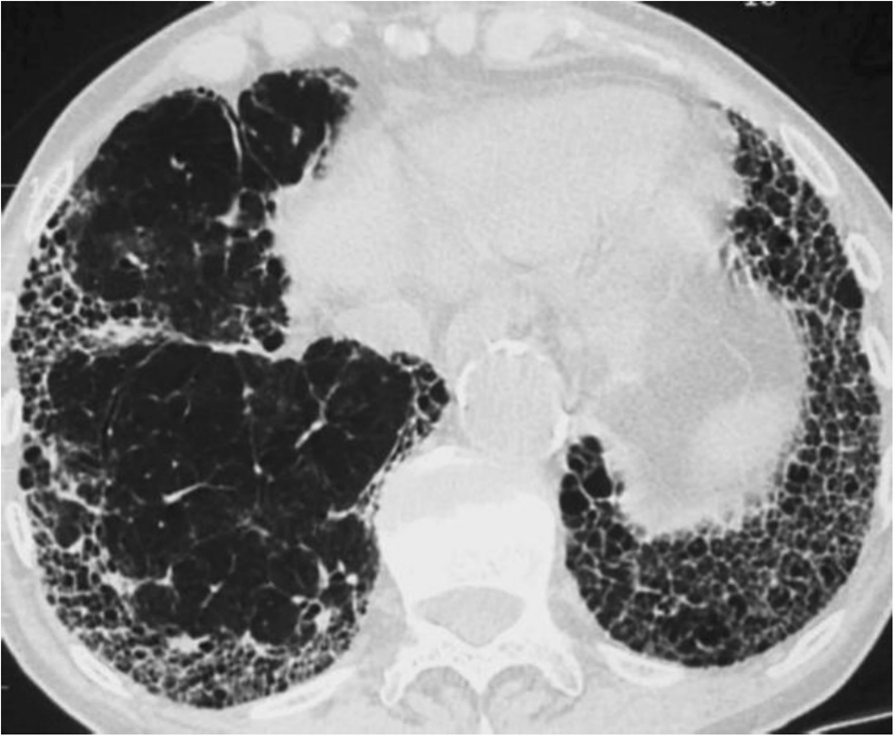
CT scan of thorax in a patient with IPF showing typical basal, sub-pleural, honeycomb shadowing and traction bronchiectasis.

Idiopathic pulmonary fibrosis (IPF) is the commonest form of idiopathic interstitial pneumonia. It is a disease of unknown aetiology which develops in the sub-pleural aspects of the posterior, basal parts of the lungs and becomes more extensive over time. On CT scan of the thorax the lungs show reticular opacities, often associated with ‘traction bronchiectasis’ and honeycombing (Figure 3). On histological examination, the features of usual interstitial pneumonia with characteristic temporal and spatial heterogeneity are evident [16,17].

It has been known for many years that cough is a clinical feature of IPF. In 1974, Scadding observed: ‘*later, with severe and extensive fibrosis cough, unproductive or with scanty mucoid expectoration, may be troublesome’*[18]. However, considerable time elapsed before the first objective study of the cough reflex in IPF was undertaken by Doherty and coworkers who demonstrated that cough sensitivity to inhaled capsaicin was increased in IPF [19]. Furthermore, by binding the chest wall of normal subjects, they demonstrated that cough was not caused by deposition of inhaled capsaicin on more central airways as a result of restricted ventilation. A subsequent study confirmed that both the two-cough and five-cough response to inhaled capsaicin was enhanced in IPF and that such patients also cough in response to substance P and bradykinin [20]. More recently, Key and colleagues used a validated cough monitor to show that patients with IPF had a 24-hour cough frequency that was similar to patients with cough hypersensitivity syndrome and significantly greater than a comparator group of asthmatics [21]. Interestingly, they also observed that cough in IPF is predominantly a daytime rather than nocturnal symptom and that there was good correlation between cough frequency and subjective measures of cough assessed by the Leicester Cough Questionnaire [21]. There is thus objective evidence from a number of sources that cough is a troublesome symptom which adversely affects quality of life in patients with IPF [22].

Superficially it appears anomalous that an interstitial lung disease, which predominantly affects the alveolar walls in the peripheral lung where sensory innervation is relatively sparse [23], should cause cough which is generally associated with diseases such as asthma, COPD and viral infections, which affect the airways where innervation is more exuberant.

A significant barrier to the methodical study of cough in IPF is that patients frequently have confounding co-morbidities which can cause or exacerbate cough. These include COPD, sinusitis, hypertension treated by angiotensin converting enzyme inhibitors and, most importantly, gasto-oesophageal reflux disease (GORD). The latter is a known cause of chronic cough [24] and it has also been postulated to be a risk factor for the development or progression of IPF [16]. The hypothesis that prolonged, small aspirations of gastric contents may be a cause of pulmonary fibrosis was proposed by Belcher in 1949 [25]. He also suggested that ‘*silent dysphagia’* was a possible cause *of ‘pulmonary lesions of obscure origin’*. This hypothesis has been given further support by recent objective studies which used 24-hour oesophageal pH monitoring and oesophageal manometry to demonstrate that up to 87% of patients with IPF have evidence of acid reflux which is often asymptomatic [26,27]. Interestingly, there are also reports of long-term stabilisation of IPF following treatment with antacid therapy [28].

A recent, preliminary study applied the technique of combined oesophageal pH and impedance monitoring, which characterises all reflux episodes regardless of pH, to show that non-acid reflux also occurs in people with IPF [29]. When combined with simultaneous, 24 hour cough recording, the study found that acid suppression therapy with proton pump inhibitors had no effect on cough and actually increased the amount of non-acid reflux. These findings, if confirmed, suggest that acid reflux, per se, is not the cause of cough in IPF. Gastric juice is a complex mixture of hydrochloric acid and enzymes such as pepsin, but it may also contain refluxate from the duodenum which is alkaline and contains bile acids and pancreatic enzymes. Potentially, gastroesophageal refluxate contains a mixture of all these components and may be gaseous as well as liquid and aspiration of this refluxate could expose respiratory epithelium to bile salts and enzymes as well as acid. In-vitro studies have shown that airway epithelial cells exposed to unconjugated bile salts produce increased levels of the fibrogenic cytokine Transforming Growth Factor-β1 [30]. There is also preliminary data to suggest that airway epithelial cells exposed to pepsin can undergo epithelial-mesenchymal transformation, a process which may be associated with the pathogenesis of IPF [31].

**Figure 4:**
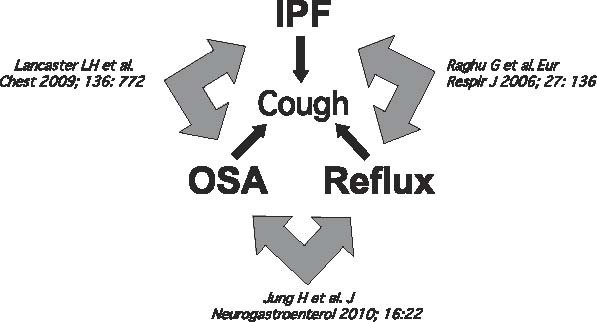
Idiopathic pulmonary fibrosis, gastroesophageal reflux and obstructive sleep apnoea: vicious triad or common associations?

The hypothesis that aspiration of gastric contents is an explanation for both the pathogenesis of IPF and its associated symptom of cough, has been confounded by a number of recent clinical and epidemiological observations. Firstly, that obstructive sleep apnoea (OSA) may be prevalent in people with IPF [32,33]; secondly, that OSA is associated with cough [34,35] and finally, the strong association between OSA with GORD [36]. It is therefore possible that the three conditions of IPF, GORD and OSA are linked in what might be described as a ‘vicious triad’ (Figure 4). However, not all evidence supports this ‘triad’ as a pathogenetic mechanism. A recent multivariate analysis in 54 patients with fibrosing interstitial lung disease found no relationship between the presence or severity of OSA and GORD and that acid reflux was no more frequent or severe in subjects with OSA compared to those without [37]. Also, a small prospective study found no difference in pepsin levels of exhaled breath condensate between patients with IPF and normal controls [38]. At present, the relationship between GORD and IPF remains uncertain and it is possible that the pattern of rapid, shallow breathing, typical of fibrotic lung in patients with IPF, might actually induce GORD [16].

A different theory for the pathogenesis of cough in IPF proposes that mechanical distortion of the larger airways by progressive interstitial fibrosis, so-called ‘traction bronchiectasis’ (Figure 3) increases cough sensitivity by altering sensory innervation of the airways [39]. Potentially, this could result from an increase in the number of rapidly adapting receptors (RARs) which respond to mechanical stimulation within the airways or, conceivably, by a destruction of nerves which inhibit cough. A recent study which tested this hypothesis compared the effect of mechanical stimulation on cough in people with IPF and controls. Interestingly, vibration of the chest wall induced a true cough reflex in the majority of people with IPF but not controls [40]. Furthermore, the two-cough response occurred most frequently on stimulation of the posterior, basal parts of the lung, where fibrosis is most extensive. Transmission of vibration, including vocal sound, is increased in pulmonary fibrosis and is the reason for the typical physical signs of enhanced tactile vocal fremitus and vocal resonance. It is therefore possible that vocalisation or indeed cough itself, might enhance cough in IPF by a mechanism of enhanced vibration and positive feedback.

**Figure 5:**
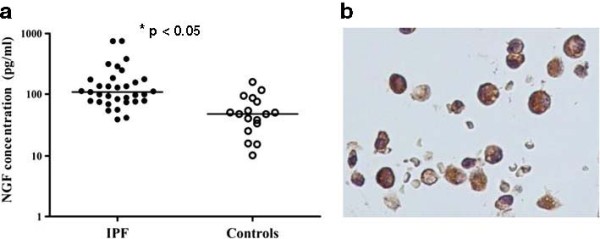
a, Concentration of Nerve Growth Factor in bronchoalveolar lavage fluid of patients with idiopathic pulmonary fibrosis and normal controls. b, Alveolar macrophages from patients with idiopathic pulmonary fibrosis immunostained for nerve growth factor. Courtesy of Dr Mat Jones (ref 41).

As with sarcoidosis, there is some evidence for increased expression of neurotrophins in the lungs of people with IPF. It has been demonstrated that induced sputum from such people contains higher levels of NGF and BDNF compared with normal controls [20] and BALF from people with IPF contains higher levels of NGF than controls (Figure 5), [41]. A single immunological study found enhanced expression of NGF and TrkA in lungs of patients with IPF compared to other interstitial lung diseases and fibroblastic foci in particular, showed immunostaining for BDNF and TrkB [42]. Thus, despite IPF being a disease of peripheral lung, these observations raise the possibility that neurotrophins, generated by the pathological process of usual interstitial pneumonia, occur in more central airways where they may influence neuronal proliferation and differentiation.

Treatment of cough in IPF remains problematic for both patients and physicians, particularly in the later stages of the condition when it may be associated with increasingly severe breathlessness. In such cases, palliative therapy using conventional anti-tussive agents such as opiate-derived preparations often proves to be of limited benefit, as does optimal pharmacological treatment of acid reflux. One small, uncontrolled, open-labeled study found that cough sensitivity to capsaicin and cough symptom score was reduced after one month of high-dose oral corticosteroids suggesting that cough in IPF should be amenable to pharmacological therapy [20]. In an open-labeled trial of oral interferon-α, five of 20 patients reported improvement in cough symptoms [43]. However, it is well known that cough is a symptom which is highly influenced by placebo effect; hence the results of small, open-labeled trials must be interpreted with caution [44].

A recent double-blind, two-treatment, two-period crossover trial comparing thalidomide with placebo demonstrated a beneficial treatment effect on cough and quality of life as determined by questionnaires and visual analogue score [45]. Whilst the authors acknowledge the limitations of the trial which include small numbers of self-referred patients with relatively mild IPF who had other possible causes of cough (70% had acid-reflux and 30% were on ACE-inhibitors for hypertension), this is the first, placebo-controlled trial of cough therapy in IPF. Thalidomide is an interesting drug that may exert its effects through immunomodulatory, anti-inflammatory or anti-angiogenic properties. However, its profile of side-effects which include constipation, bradycardia, dizziness and in the longer term, peripheral neuropathy, suggest it may have a more direct effect on airway nerves. Certainly, the potential anti-tussive effects of thalidomide warrant further investigation.

At the present time, physicians are all too often reduced to treating the debilitating cough of IPF with opiates as part of an end-of-life pathway. There is thus an urgent need for better treatments which can only be developed through our better understanding of the cough reflex in IPF.

## Competing interest

The author declares that he has no competing interests.
